# Sea Sickness

**Published:** 1878-02

**Authors:** 


### SEA SICKNESS

Is caused in great part by the confusing effect which the tossing on the water has upon the brain, and multitudes of ways have been pursued for avoiding or at least mitigating this annoyance. The best plan is to let it have its course and rid the system of that excess of bile which is almost always present in this over-eating age; the general health rarely fails to be greatly improved by it, although in very rare cases, perhaps not over one in a million, dies under the effects of the long continued and exhausting retching. If a person will lie down with the eyes closed, and not allow the head for an instant to be raised from the pillow there is an almost entire exemption from nausea and other discomforts, but the result of this course is that it will be necessary to keep a-bed during the entire voyage; the effort should be to shorten the sickness and get rid of it as soon as possible, and this is best done by not lying down at all, but resolutely keeping on the feet on deck, in the open air, if the weather permits, that is, if it is not raining; this requires moral courage and some considerable force of will and character, but it seldom fails to abridge the period of sea-sickness, sometimes to confine it to a few hours duration and then the remainder of the voyage can be enjoyed as it ought to be.

The tendency to nausea on ship-board is abated somewhat by any stimulus which acts decidedly on the nervous system, such as chloroform, brandy, opiates, etc. Irritants, such as the strongest spices, abate nausea; so will great mental emotions, in short, any thing which draws off the attention of the mind. No person can get sea-sick if the ship is on fire, nor will a person who is drunk. A brisk purgative is good just before going on board or a dose of medicine taken the night before. Still the wisest, most healthful and most expeditious method of meeting sea-sickness is to avoid all preventitives, all medicines, and manfully determine to keep upon your feet and let it do its worst.

In this connection space may be given to

